# Dynamic variation of CD5 surface expression levels within individual chronic lymphocytic leukemia clones

**DOI:** 10.1016/j.exphem.2016.09.010

**Published:** 2017-02

**Authors:** Rachael J.M. Bashford-Rogers, Anne L. Palser, Clare Hodkinson, Joanna Baxter, George A. Follows, George S. Vassiliou, Paul Kellam

**Affiliations:** a Department of Medicine, University of Cambridge, Cambridge Biomedical Campus, Cambridge, UK; b Wellcome Trust Sanger Institute, Wellcome Trust Genome Campus, Hinxton, Cambridge, UK; c Cambridge Blood and Stem Cell Biobank, University of Cambridge, Department of Haematology, National Health Service Blood and Transplant Cambridge Centre, Cambridge, UK; d Department of Haematology, Addenbrooke's Hospital, Cambridge, UK; e Research Department of Infection, Division of Infection and Immunity, University College London, London, UK

## Abstract

Chronic lymphocytic leukemia (CLL) is characterized by the accumulation of clonally derived mature CD5^high^ B cells; however, the cellular origin of CLL is still unknown. Patients with CLL also harbor variable numbers of CD5^low^ B cells, but the clonal relationship of these cells to the bulk disease is unknown and can have important implications for monitoring, treating, and understanding the biology of CLL. Here, we use B-cell receptors (BCRs) as molecular barcodes to first show by single-cell BCR sequencing that the great majority of CD5^low^ B cells in the blood of CLL patients are clonally related to CD5^high^ CLL B cells. We investigate whether CD5 state switching was likely to occur continuously as a common event or as a rare event in CLL by tracking somatic BCR mutations in bulk CLL B cells and using them to reconstruct the phylogenetic relationships and evolutionary history of the CLL in four patients. Using statistical methods, we show that there is no parsimonious route from a single or low number of CD5^low^ switch events to the CD5^high^ population, but rather, large-scale and/or dynamic switching between these CD5 states is the most likely explanation. The overlapping BCR repertoires between CD5^high^ and CD5^low^ cells from CLL patient peripheral blood reveal that CLL exists in a continuum of CD5 expression. The major proportion of CD5^low^ B cells in patients are leukemic, thus identifying CD5^low^ B cells as an important component of CLL, with implications for CLL pathogenesis, clinical monitoring, and the development of anti-CD5-directed therapies.

## Highlights

• Surface CD5 expression heterogeneity exists within CLL clones.
• The majority of CD5^low^ B cells in CLL are part of the leukemic clone.
• Large-scale dynamic relationships exist between CD5^high^ and CD5^low^ B cells in CLL.
• This phenomenon has implications for disease biology and treatment.

Chronic lymphocytic leukemia (CLL) is characterized by the accumulation of clonally derived mature CD5^+^CD19^+^CD23^+^CD20^+^ B cells in the blood, bone marrow, and secondary lymphoid organs [1]. CD5 is a glycoprotein normally found on T cells and a subset of immunoglobulin M (IgM)-secreting B cells known as B-1a cells [2], as well as regulatory B cells [3], but not on the majority of peripheral blood (PB) B cells in healthy adults. Although expanded B-cell populations in CLL patients typically have high CD5 expression, the cellular origin of CLL is still unknown. CD5^+^ CLL B cells show similar gene expression patterns to the healthy CD5^+^ B-1a B cells [4], but differ significantly from these cells with regard to other surface markers, exhibiting features of either activation or anergy after antigenic interactions [5]. As a result, there is still ambiguity as to whether CD5 is a marker of activation rather than of B-cell subtype [6]. The fluidity between CD5^+^ and CD5^–^ states in normal B cells is demonstrated in vitro by the induction of CD5 cell-surface expression in B2-B cells by stimuli such as anti-IgM antibodies and phorbol 12-myristate-13-acetate 7, 8 and by downregulation of CD5 in CD5^+^ B-1a B cells by exposure to cytokines [9].

CLL patients regularly harbor CD5^low^ B cells, but the relationship of these cells to the leukemic cell bulk is unknown. If CD5^low^ B cells formed part of the CLL clone, then this would have important implications for monitoring, treating, and understanding the biology of CLL. Because CD5 expression is commonly used as a marker for CLL, the presence of CD5^low^ tumor B-cell populations would suggest that the “true” tumor load in patients is underestimated. Moreover, the identification of a CD5^low^ subpopulation in CLL would have significant implications for the development of therapeutic anti-CD5 monoclonal antibodies for CLL 10, 11, 12. Furthermore, the study of the cellular origin and molecular pathogenesis of CLL would benefit from a better understanding of the diversity of clonal B cells and the role of any CD5^low^ subpopulation [4] given that studies normally focus on the CD5^+/high^ B-cell populations [13]. Here, we first demonstrate the heterogeneity of CD5 expression within CLL clones from individual patients and identify dynamic relationships between CLL cells with high and low CD5 expression. We then show for the first time that there exists a large-scale dynamic relationship between CD5^high^ and CD5^low^ B-cell populations in CLL, a phenomenon with implications for disease biology and treatment.

## Methods

### Patient samples

PB mononuclear cells (PBMCs) were isolated from 10 mL of whole blood from four healthy volunteers and four CLL patients using Ficoll gradients (GE Healthcare) for bulk-sequencing experiments. Single-cell and bulk-cell flow sorting were performed using CD20-FITC, CD19-PE, CD5-APC, and IgG-V450 (BD Biosciences) and Aqua (for live-dead cell detection, Invitrogen) into 96-well plates from 1.5–1.9 × 10^6^ frozen PBMCs per individual. Total RNA was isolated using TRIzol (Invitrogen) and purified using the RNeasy Mini Kit (Qiagen) including on-column DNase digestion according to the manufacturer's instructions. Research was approved by the relevant institutional review boards and ethics committees (07/MRE05/44). Patient information is listed in Supplementary Table E1, Supplementary Table E2, Supplementary Table E3 (online only, available at www.exphem.org).

### B-cell receptor (BCR) amplification and sequencing

Reverse transcriptase polymerase chain reactions were performed using FR1 primers as described previously [14]. MiSeq libraries were prepared using Illumina protocols and sequenced using 300-bp paired-end MiSeq (Illumina). MiSeq reads were filtered for base quality (median >32) using QUASR [15] and paired-end reads merged if they contained identical overlapping regions of >65 bp or otherwise discarded. Non-Ig sequences were removed and only reads with significant similarity to reference Ig heavy chain variable (IgHV) genes in the international ImMunoGeneTics information system (IMGT) database [16] by BLAST [17] were retained (<1 × 10^−10^ E-value). Primer sequences were trimmed from reads and sequences were retained for analysis only if both forward and reverse primer sequences were identified and sequence lengths were greater than 240 bp for MiSeq. Single-cell BCR sequencing was performed as described previously [14], with the number of PCR cycles increased to 50 and the PCR product undergoing Sanger sequencing. Only wells with a single BCR Sanger sequencing signal present were used in downstream analyses.

### Network assembly and analysis

The network generation algorithm and network properties were calculated as in Bashford-Rogers et al. [14]. Briefly, each vertex represents a unique sequence in which relative vertex size is proportional to the number of identical sequence reads. Edges were generated between vertices that differed by single-nucleotide, non-indel differences and clusters were collections of related, connected vertices. Phylogenetic analyses were performed by alignment using Mafft [18] and maximum parsimony tree fitting using Paup* [19].

### Testing single versus common CD5^+^/CD^–^-switching event hypotheses

The hypothesis that a population of B-CLL clones with distinct BCR sequences switched CD5^high^/CD5^low^ states rather than a single switching event can be tested statistically by calculating the probability that an overlap of BCR sequences between CD5^high^ and CD5^low^ samples can happen by chance given a single shared sequence. If the BCR sequence length is *l*, and nucleotide distance from the central BCR is *d*, and any position can become mutated to any one of three other bases, then the number of potential mutational combinations is defined by:

$$Number\;of\;combinations\;of\;mutations=\;\left(\frac{l!}{\;\left(l-d\right)!d!}\right){\cdot 3}^{d}$$

The hypergeometric test was used to determine the probability of observing, between CD5^high^ and CD5^low^ samples, equal or greater BCR sequence overlap than what would be expected by chance.

To test whether the CD5^high^ and CD5^low^ CLL B-cell BCR populations had a tendency to co-cluster, the nucleotide distances between any two sequences within or between the CD5^high^ and CD5^low^ CLL B-cell BCR populations was determined. The ratio between the mean nucleotide distances within versus between the CD5^high^ and CD5^low^ CLL B-cell BCR populations was calculated. By calculating this ratio from representatively sized random samples of the data (bootstrapping), the *p* value that the CD5^high^ and CD5^low^ CLL B-cell BCR populations were distinctly clusters rather than random mixing was determined. Here we used 1000 bootstraps of the data to calculate the *p* value.

## Results

### Single-cell sequencing reveals that CLL B cells have heterogeneous CD5 surface expression

**Supplementary Figure E1:**
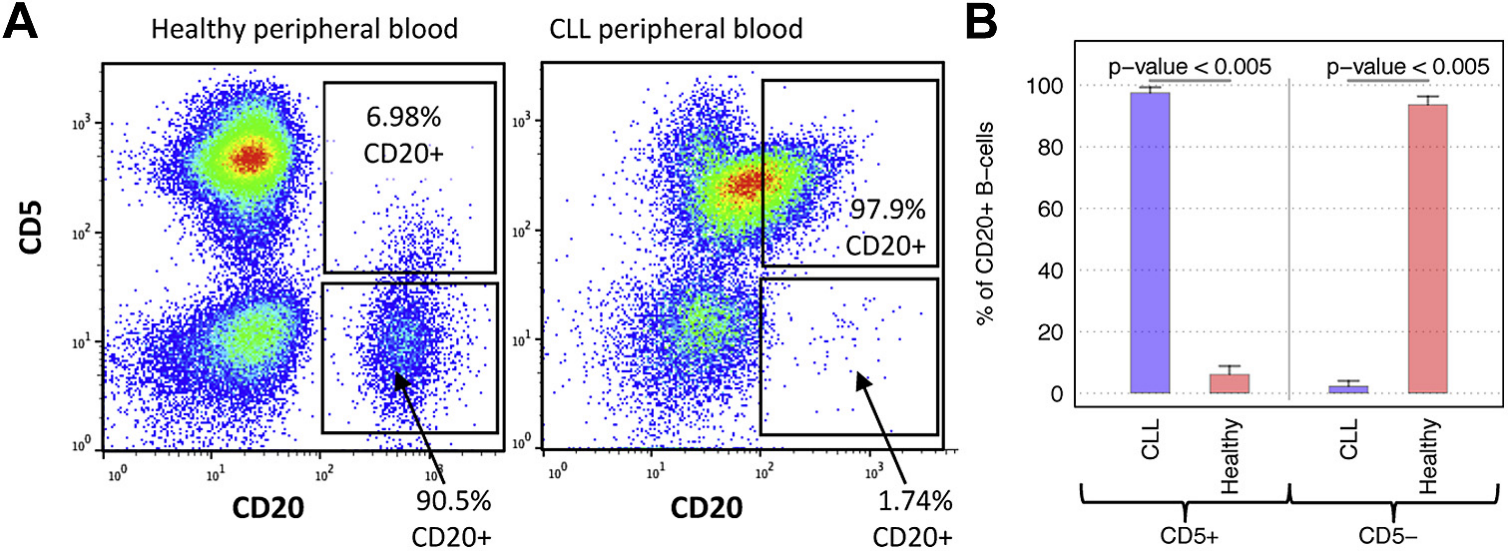
Comparative analysis of CD5^high^ and CD5^low^ B-cell populations in CLL patients and healthy individuals. (**A**) Peripheral blood mononuclear cell-surface expression of CD5 and CD20. CLL patients showed a higher proportion of CD20^+^ B cells with high CD5 cell-surface expression compared to healthy individual samples. One representative sample is shown for each. (**B**) The percentages of CD5^high^ and CD5^low^ B cells (CD20^+^) in four CLL patients and four healthy individuals, with unpaired *t*-test *p* values indicated above sample groups. Error bars indicate mean + SD.

We first sought to determine whether CD5^low^ B cells can form part of the CLL clone in patients and to describe their relationship to CD5^high^ cells from the same CLL. Flow cytometry and cell sorting were performed on PB from four CLL patients (white blood counts ranged from 69.1 × 10^9^ to 102.4 × 10^9^/L) and four healthy age-matched individuals (summarized in Supplementary Table E1, online only, available at www.exphem.org). In agreement with previous studies [1], we found considerably higher proportions of CD5^high^ B cells in the PB of all four CLL patients studied (>96% of CD20^+^ B cells) compared with healthy, age-matched controls (4.6–7.4%, *p* < 0.005; Supplementary Fig. E1 and Supplementary Table E4, online only, available at www.exphem.org). We also identified in all patients detectable populations of B cells with no or low-level expression of surface CD5 (∼1.5-3.2% of B cells).

**Figure 1:**
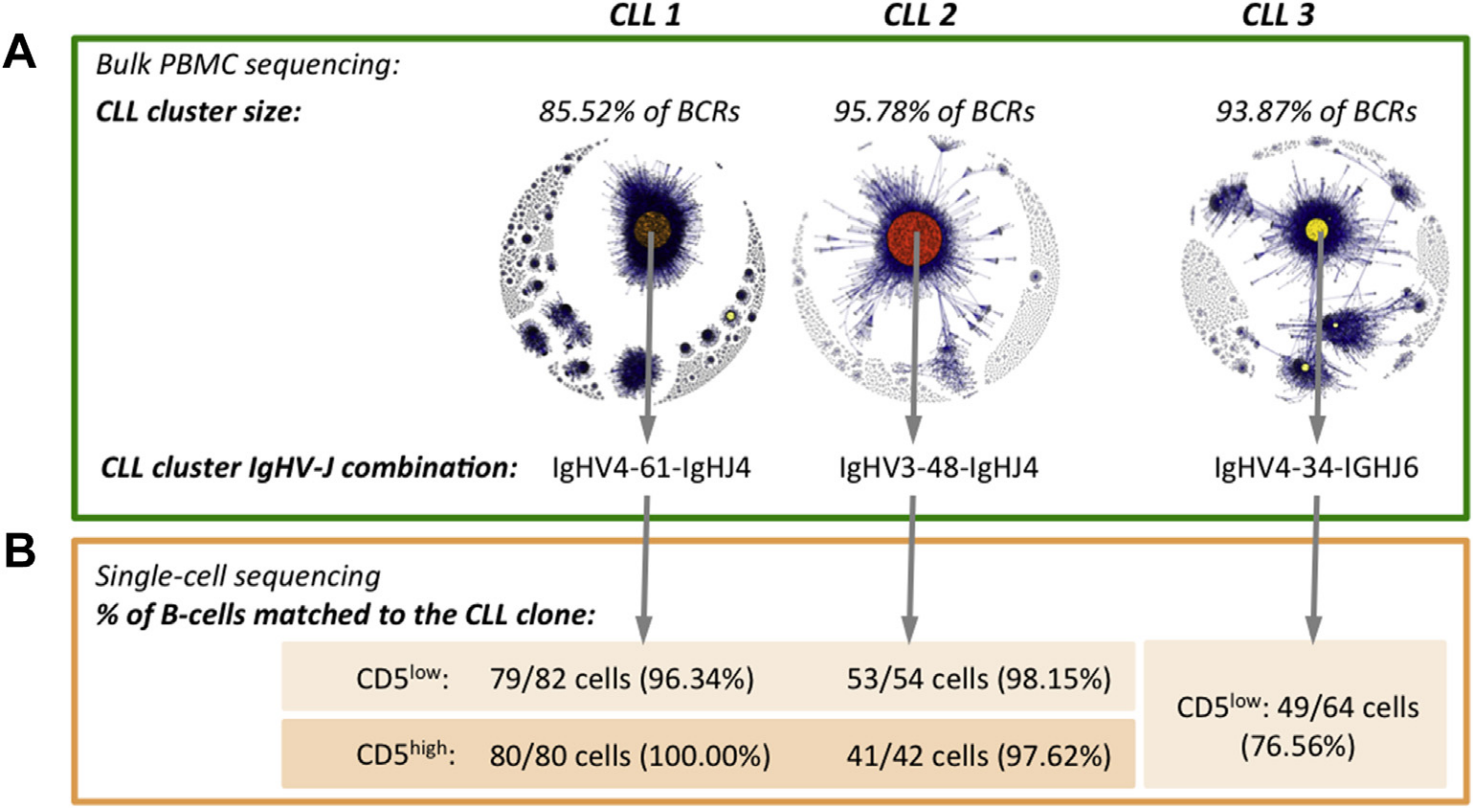
Comparative analysis of CD5^high^ and CD5^low^ B-cell populations in CLL patients and healthy individuals. (**A**) Bulk-cell total PBMC BCR sequencing networks for CLL patients 1-3 with corresponding maximum cluster sizes and IgHV-J gene usage. Samples yielded 112,722, 222,801, and 151,777 BCR sequences for CLL patients 1, 2, and 3, respectively (after filtering for Ig similarity, length, and primer sequences removal according to Bashford-Rogers et al. [14]). Sequencing networks are presented such that each vertex represents a unique BCR sequence in which relative vertex size is proportional to the number of identical sequence reads. Edges were generated between vertices that differed by single-nucleotide, non-indel differences and clusters were collections of related, connected vertices. The largest cluster sizes (CLL clusters) and corresponding IgHV-J combinations are indicated above and below the networks, respectively. (**B**) Single-cell analysis of CD5^high^ or CD5^low^ B-cell populations: single-cell BCR sequencing of CD5^high^ and CD5^low^ B cells from CLL patients 1, 2, and 3 was used to determine the frequencies of CLL cells in each B-cell subset. The number and percentage of CD5^high^ or CD5^low^ B cells expressing BCRs matching (i.e., maximum of 3 bp difference from the dominant CLL BCR sequence) the CLL clone for each CLL patient are indicated.

To study these CD5^low^ B cells, we first determined the clonal BCR sequences present in total PB lymphocytes, which were dominated by the CLL lymphocytes, and used these to mine identical sequences for clonal CLL cells in flow-sorted CD5^high^/CD5^low^ B-cell populations at the single-cell level. BCRs are generated during B-cell development by site-specific DNA recombination of V, (D), and J genes, with nontemplate additions and deletions between genes. Therefore, each B-cell clone expresses a unique BCR sequence and we have used BCR sequencing previously to identify B cells originating from the same clone [14]. Next-generation sequencing of BCRs from cDNA of total PB B-cell populations from three CLL patients (two untreated and one previously treated with chlorambucil/rituximab; Supplementary Table E1, Supplementary Table E2, online only, available at www.exphem.org) yielded between 112,722 and 222,801 BCR sequences (after filtering for Ig similarity, length, and primer sequences removal according to Bashford-Rogers et al. [14]). Network analysis was applied to these PBMC bulk-cell sequencing datasets to identify CLL clusters representing groups of highly related BCR sequences [14], with each CLL patient sample exhibiting enlarged clusters of related sequences (identical IgHV-D-J rearrangements and joining regions), representing >85% of all BCR sequences (Fig. 1A). These enlarged clusters corresponded to the BCRs expressed by the expanded CLL clone, similar to previous studies [14]. The IgHV-J combinations for these clusters were defined as IGHV4-34-IGHJ6, IGHV4-61-IGHJ4, and IGHV3-48-IGHJ4 for CLL patients 1, 2, and 3 respectively (Fig. 1Bi).

**Supplementary Figure E2:**
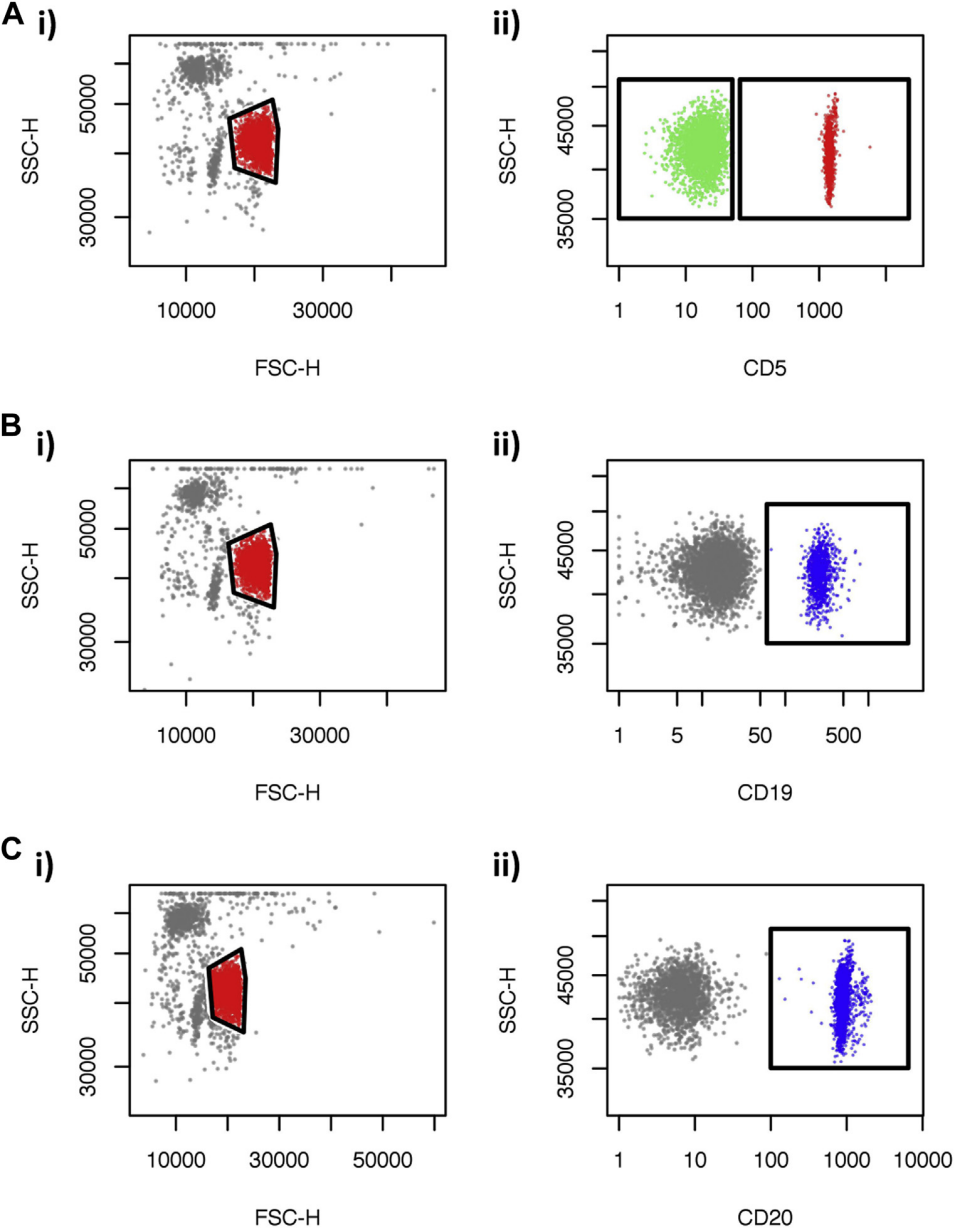
Optimizing gates for single-cell FACS sorting using beads. Polystyrene microparticle beads were mixed with the fluorochrome-conjugated human antibodies to optimize fluorescence gating settings for flow cytometric single-cell sorting of CD5^high^ and CD5^low^ B cells. After gating of monomeric microparticle beads, indicated by the *red* gates in part (**i**) in each panel, the (**A**) CD5^high^ and CD5^low^ gates (*red* and *green*, respectively) were set around the positive and negative control CD5 microparticle beads, (**B**) CD19 positive gates (*blue*), and (**C**) CD20 positive gates (*blue*).

**Supplementary Figure E3:**
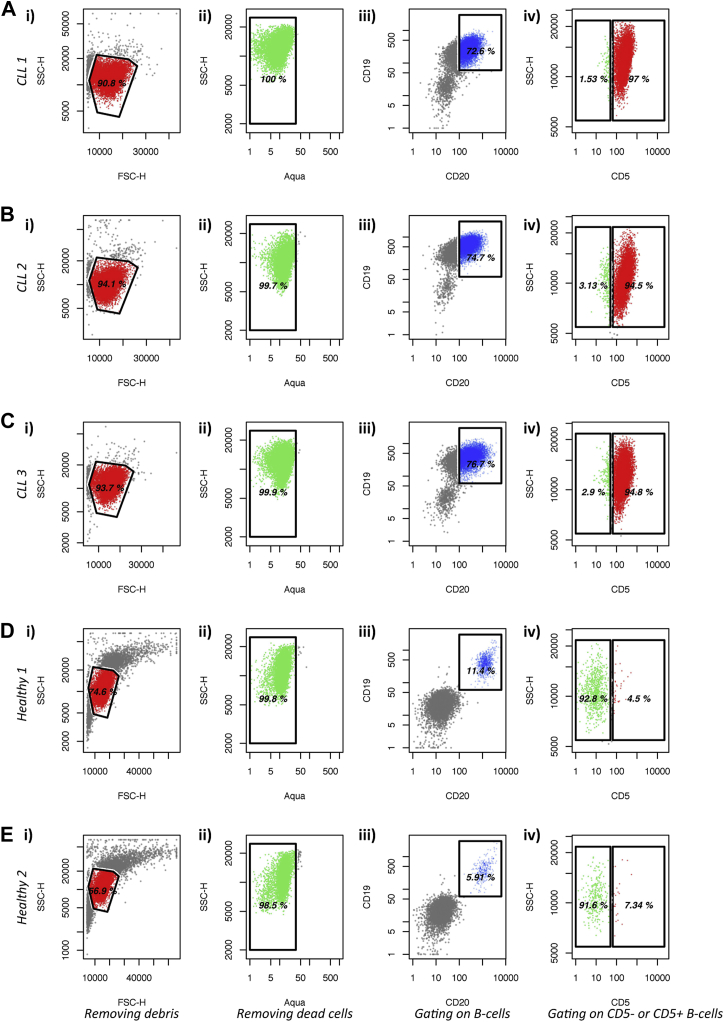
Single-cell flow sorting of CD5^high^ and CD5^low^ B cells. The gating strategy used for identification of CD5^high^ and CD5^low^ mature peripheral blood B cells in the single-cell experiments for (**A**) CLL patient 1, (**B**) CLL patient 2, (**C**) CLL patient 3, (**D**) healthy individual 1, and (**E**) healthy individual 2. After exclusion of debris using light scatter features of leukocytes (panels (**i**)) and exclusion of dead cells (panels (**ii**)), mature B cells were identified as CD19^+^CD20^+^ (panels (**iii**)). Events captured in the *red* gate in panels (**iv**) were identified as CD5^high^, and those captured in *green* gate were identified as CD5^low^. Gates were chosen based on bead experiments (Supplementary Fig. E1).

After determining the CLL BCR sequences in bulk PB samples, we confirmed the presence of CLL cells in both the CD5^high^ and CD5^low^ B-cell subsets using single-cell BCR sequencing. CD5^high^ and CD5^low^ single B cells were sorted into 96-well plates (gating strategies in Supplementary Figure E2, Supplementary Figure E3, online only, available at www.exphem.org), where single-cell BCR amplification and sequencing was performed. Successful amplification of 42–82 single CD5^high^ or CD5^low^ cells per fluorescence-activated cell sort was achieved per patient. Expectedly, 97.26–100% of single CD5^high^ B cells expressed the CLL clonotypic BCR sequence in each patient. Interestingly, 76.56–98.15% of CD5^low^ BCR sequences also matched to the CLL clone sequence (i.e., the sequence was identical to a BCR present in the bulk CLL clonal cluster; Fig. 1B; Supplementary Table E5, online only, available at www.exphem.org). Only one BCR sequence was detected in each single cell/well in all cases. Because putative co-occupancy of the same well by both a CD5^low^ non-CLL cell with a distinct BCR and a CD5^high^ CLL cell with a CLL BCR would produce two unique BCR sequences, the detection of clonotypic cells in the CD5^low^ wells indicates that these cells truly form part of the CLL clone. This demonstrates that the majority of CD5^low^ B cells form part of the CLL clone, at least in the three patients studied here.

### Population structures are shared between CD5^low^ and CD5^high^ B-cell populations in CLL patients, but not in healthy individuals

**Supplementary Figure E4:**
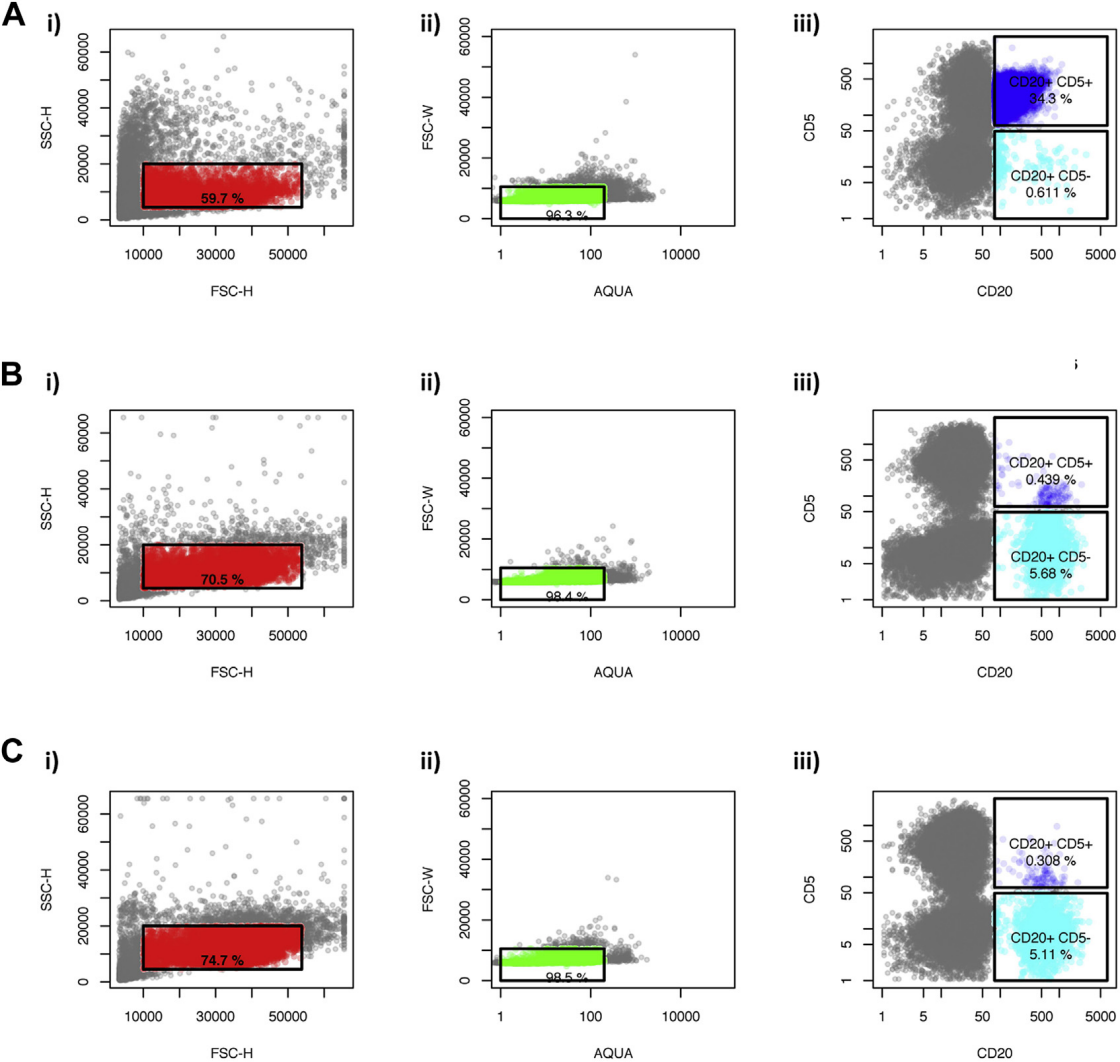
Bulk-cell flow sorting of CD5^high^ and CD5^low^ B cells. The gating strategy used for identification of CD5^high^ and CD5^low^ mature peripheral blood B cells in the bulk-cell experiments for (**A**) CLL patient 4, (**B**) healthy individual 3, and (**C**) healthy individual 4. After exclusion of debris using light scatter features of leukocytes (panels (**i**)) and exclusion of dead cells (panels (**ii**)), mature B cells were identified as CD20^+^ (panels (**iii**)). Events captured in the *dark-blue* gate in panels (**iii**) were identified as CD5^high^, and those captured in *light-blue* gate were identified as CD5^low^.

**Supplementary Figure E5:**
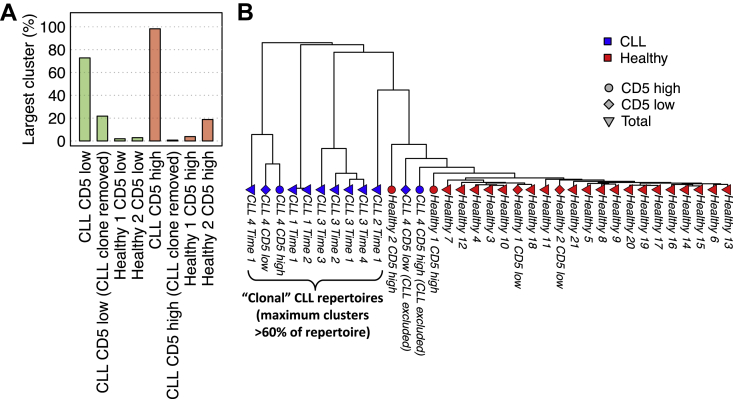
Analysis A.of the nonleukemic counterpart of CD5^+/–^ B-cell populations in CLL patients. (**A**) Comparisons of the largest cluster sizes (as a percentage of total reads) and (**B**) hierarchical clustering of IgHV-J gene usage similarities between CD5^high^ (*red*) and CD5^low^ (*green*) B-cell populations for two healthy individuals and CLL patient 4 (either for all reads in the sample, or all reads that were unrelated to the CLL clone, defined as >30 bp differences and/or >10 gaps within an alignment from the CLL clonal sequence). The CD5^high^ and CD5^low^ samples from patient 4 have increased clonality compared to healthy individuals (Supplementary Fig. E5A). However when the sequences related to the CLL clone are removed from the CD5^high^ and CD5^low^ samples (i.e., the nonleukemic counterpart of the repertoire), there is no significant difference in clonality of V-J gene usage frequencies. Both CD5^high^ and CD5^low^ samples from CLL patient 4, when excluding the CLL clone, cluster together with the healthy individuals.

**Figure 2:**
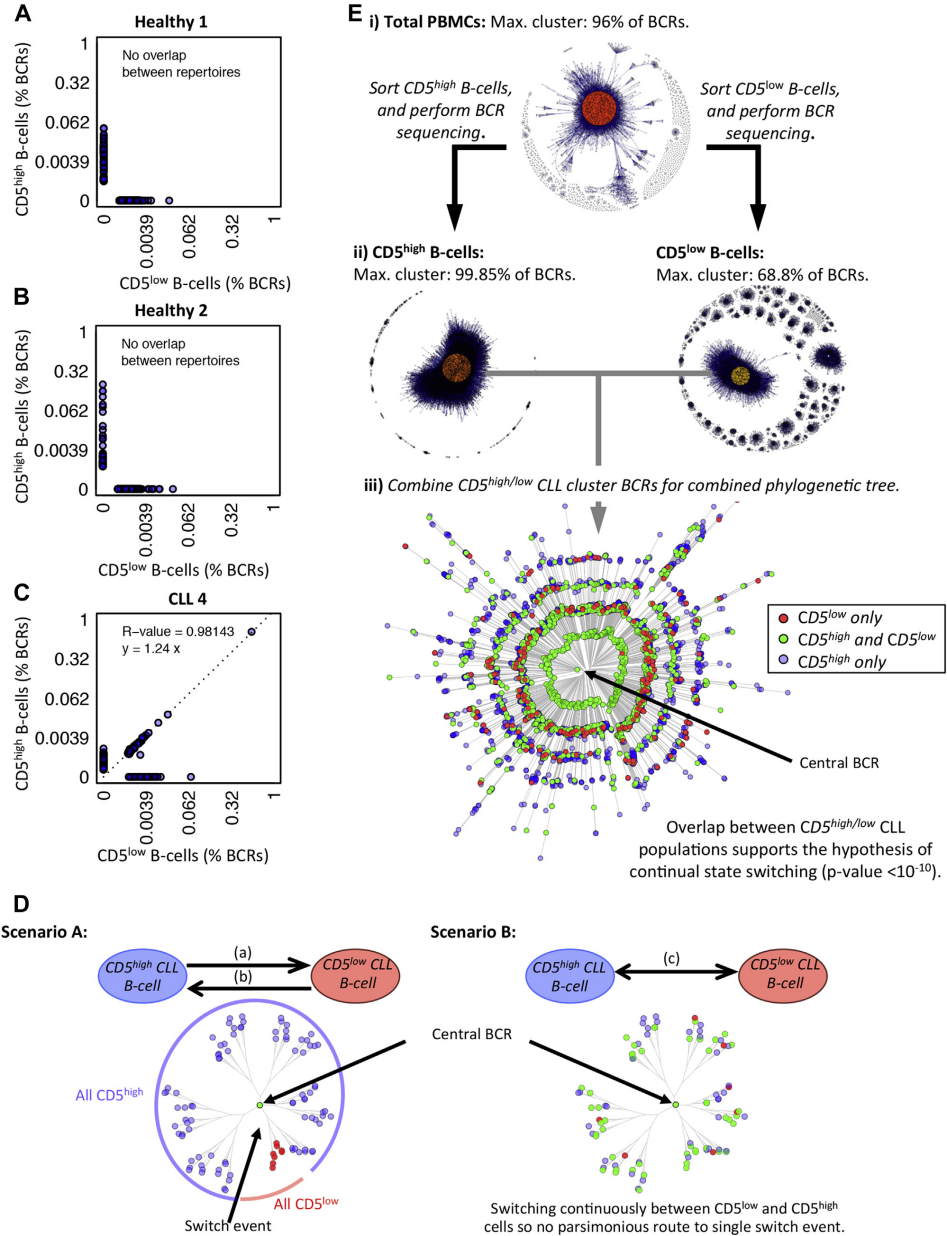
Comparison of CD5^high^ and CD5^low^ B-cell populations. Shown are plots of the frequencies of individual BCRs between CD5^low^ and CD5^high^ B-cell populations for healthy patient 1 (**A**), healthy patient 2 (**B**), and CLL patient 4 (**C**) as determined by sequencing the BCR repertoires using MiSeq. For CLL patient 4, in whom there is an overlap between CD5^low^ and CD5^high^ samples, the least-squares regression line equation and *R*^2^ value was determined for all BCRs, as indicated. (**D**) Theoretical model of CD5-state switching: unidirectional switch from CD5^high^ to CD5^low^ (scenario A), unidirectional switch from CD5^high^ to CD5^low^ (scenario B), and bidirectional dynamics switching/alternating between states (scenario C). These processes can be either a rare event (occurring only from a single or low number of CLL B cells) or a common/continuous event (occurring from a large percentage of the CLL B-cell population). (**E**) Determining the dynamics of CD5 state-switching in CLL patient 4: BCR sequence networks for total PBMCs (**Ei**) and BCR sequence networks for separated CD5^high^ B cells (**Eii, left**) and CD5^low^ B cells (**Eii, right**). The dominant cluster sizes are indicated above each plot. (**Eiii**) Combined maximum parsimony phylogenetic tree of the CLL cluster generated using the combined CLL BCR sequences from the separated CD5^high^ and CD5^low^ B-cell populations from (**Eii**). The branch lengths are proportional to the number of base differences from the central BCR sequence (evolutionary distance) and the resulting tree tips are colored red if the BCR was observed in CD5^low^ B cells only, blue if observed in CD5^high^ B cells only, and green if observed in both CD5^low^ and CD5^high^ B cells. Bootstrapping was performed to evaluate the reproducibility of the trees, showing strong tree support (>90% certainty for all branches). The overlap between CD5^low^ and CD5^high^ CLL BCR populations was significantly greater than that expected by a rare CD5 state-switching event originating from the central BCR (*p* < 10^−10^).

Having established that CLL cells with identical BCRs are present in both the CD5^high^ and CD5^low^ B-cell subsets by single-cell analysis, we investigated the relationships between these two subsets. CD5^high^ and CD5^low^ B cells (all CD20^+^) from CLL patient 4 and two healthy individuals were cell sorted, yielding >10,000 B cells per sample, and high-throughput BCR sequencing was performed (generating between 11,375 and 397,469 reads; Supplementary Fig. E4 and Supplementary Table E5, online only, available at www.exphem.org), along with BCR sequencing of the unsorted B-cell population. There was no overlap of identical BCR sequences observed between the CD5^high^ and CD5^low^ B-cell populations between the two healthy individuals (Fig. 2A and 2B); however, significant overlap was observed between the CD5^high^ and CD5^low^ B-cell populations in the CLL patient (Fig. 2C). Indeed, BCR sequence network analysis showed that the expanded cluster in the total (unsorted) PBMCs (comprising 99.85% of total BCR sequences with the [IGHV3-7*02-IGHD3-10*02-IGHJ4*02] rearrangement, Fig. 2Ei) corresponded to the same clones in the expanded clusters in both the CD5^high^ and CD5^low^ B-cell samples (comprising 92.27% and 68.8% of total BCR sequences, respectively, Fig. 2Eii). Expanded clones were not observed in the healthy B-cell samples (Supplementary Table E6, online only, available at www.exphem.org). The subclonal CLL BCR frequencies were highly correlated between the CD5^high^ and CD5^low^ subsets (*R*^2^ = 0.98143, Fig. 2C), revealing similar population structures between the two CLL B-cell populations. This suggests that the B-cell population structure is shared between the CD5^high^ and CD5^low^ B-cell subsets in CLL, unlike the CD5^high^ and CD5^low^ B-cell subsets in healthy individuals that exhibit unique B-cell populations. We observed that this difference in repertoire in CLL is due to the leukemic clone, in which there are no significant differences in repertoire structure in either CD5^high^ or CD5^low^ subsets once CLL clonal BCRs were removed from the repertoires (Supplementary Fig. E5, online only, available at www.exphem.org).

### BCR phylogenetics reveals co-evolution of the CD5^low^ and CD5^high^ CLL B-cell populations

**Supplementary Figure E6:**
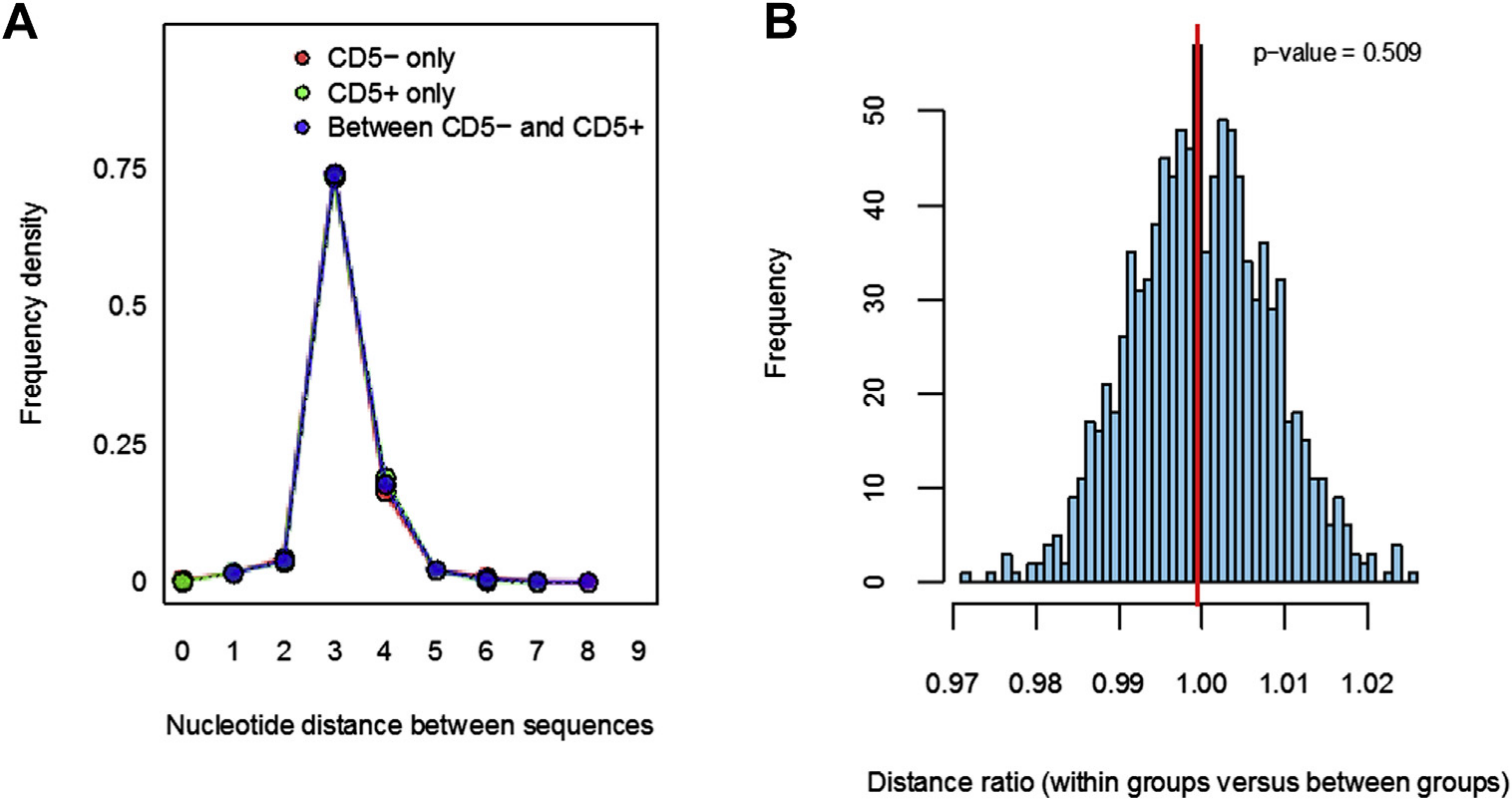
Testing the sub-clustering of CD5^high^ and CD5^low^ BCR sequences in the CLL BCR sequences in CLL patient 4. (**A**) Histogram of the nucleotide distances between any two sequences within the CD5^low^ (*red*) or CD5^high^ (*green*) or between the sequences of the CD5^high^ and CD5^low^ subsets (*blue*). The three subsets showed indistinguishable distributions of nucleotide distance distributions suggesting that the CD5^high^ and CD5^low^ CLL B cells are co-clustered. (**B**) To determine whether the CD5^low^ and CD5^high^ sequences subcluster into distinct groups on the phylogenetic tree (where the null hypothesis is that they are randomly distributed on the phylogenetic tree), the distances were randomly sampled at the same proportions, and the histogram of distance ratios of within groups versus between groups was calculated for each iteration. The *p* value for which the observed data distance ratio was nonrandom is not significant (bootstrapped *p* = 0.509), suggesting that the null hypothesis should be accepted and the sequences that generate the pairwise distances are randomly distributed on the tree (i.e., there is no parsimonious route to a single or low number of switch events).

The shared clonal structure between CD5^high^ and CD5^low^ CLL B cells suggests that either CLL B cells start from a CD5^high^ state and downregulate surface CD5, start from a CD5^low^ state and upregulate surface CD5, or alternate between the two states (Fig. 2D). To understand whether CD5 state switching was likely to occur continuously (a common event, randomly distributed over a phylogeny of CD5^high^ and CD5^low^ BCR sequences) or as a rare event (at a single point in CLL evolution, distributed along single lineages of CD5^high^ or CD5^low^ BCR sequences), we determined phylogenetic pattern of the CD5^high^ and CD5^low^ BCR sequences and the probability that an overlap of unique BCR sequences between the CD5^low^ and CD5^high^ samples can happen by chance after a single CD5 state-switching event. The accumulation of mutations during CLL clonal expansion leading to intraclonal diversification within the BCR allowed us to reconstruct the phylogenetic relationships. Maximum parsimony trees were fitted using the CLL BCR sequences (i.e., BCRs represented in the largest network cluster) from each of the sorted CD5^low^ and CD5^high^ B-cell subsets (Fig. 2Eiii). The central BCR in both CD5^high^ and CD5^low^ phylogenetic trees were identical, and was the most frequently observed BCR in both samples (comprising 85.6% and 85.7% of total CLL BCRs respectively). The star-like structures of both trees suggest that the original CLL clones in both the CD5^high^ and CD5^low^ B-cell subsets emerged from a single common ancestor, represented by the same central BCR [20]. When fitting a maximum parsimony tree of the combined CD5^low^ and CD5^high^ CLL BCR sequences (Fig. 2Eiii), substantial overlap between the BCR sequences was observed in two B-cell subsets. In fact, significantly greater overlap between the CD5^high^ and CD5^low^ CLL BCRs was observed than would be expected if the CD5 state switch occurred only from cells expressing the central CD5^high^ CLL BCR or vice versa (hypergeometric test *p* < 10^−10^; Supplementary Table E7, online only, available from www.exphem.org). If switching from CD5^high^ to CD5^low^ or from CD5^low^ to CD5^high^ were one-directional and fixed/stabilized afterward, then the merged phylogenies would be dominated by lineages of enduring BCRs fixed for CD5^high^ or CD5^low^ cells. Furthermore, the CD5^low^ and CD5^high^ sequences do not subcluster significantly into distinct groups on the phylogenetic tree (*p* = 0.509; Supplementary Fig. E6, online only, available from www.exphem.org), suggesting that there is no parsimonious route to a single or low number of switch events followed by fixed BCR sequence lineages in CD5^high^ and CD5^low^ CLL. This study supports a hypothesis that CD5 state switching is likely to occur as a common event and continuous switching may occur, although not at an equal frequency as CD5^high^/CD5^low^ ratios in CLL do not tend to 1. Decreased CD5 expression corresponds with the normal development of B cells to plasma cells [21] and it is possible that the CD5^low^ CLL population represents further differentiated CLL subclones.

## Conclusions

**Supplementary Figure E7:**
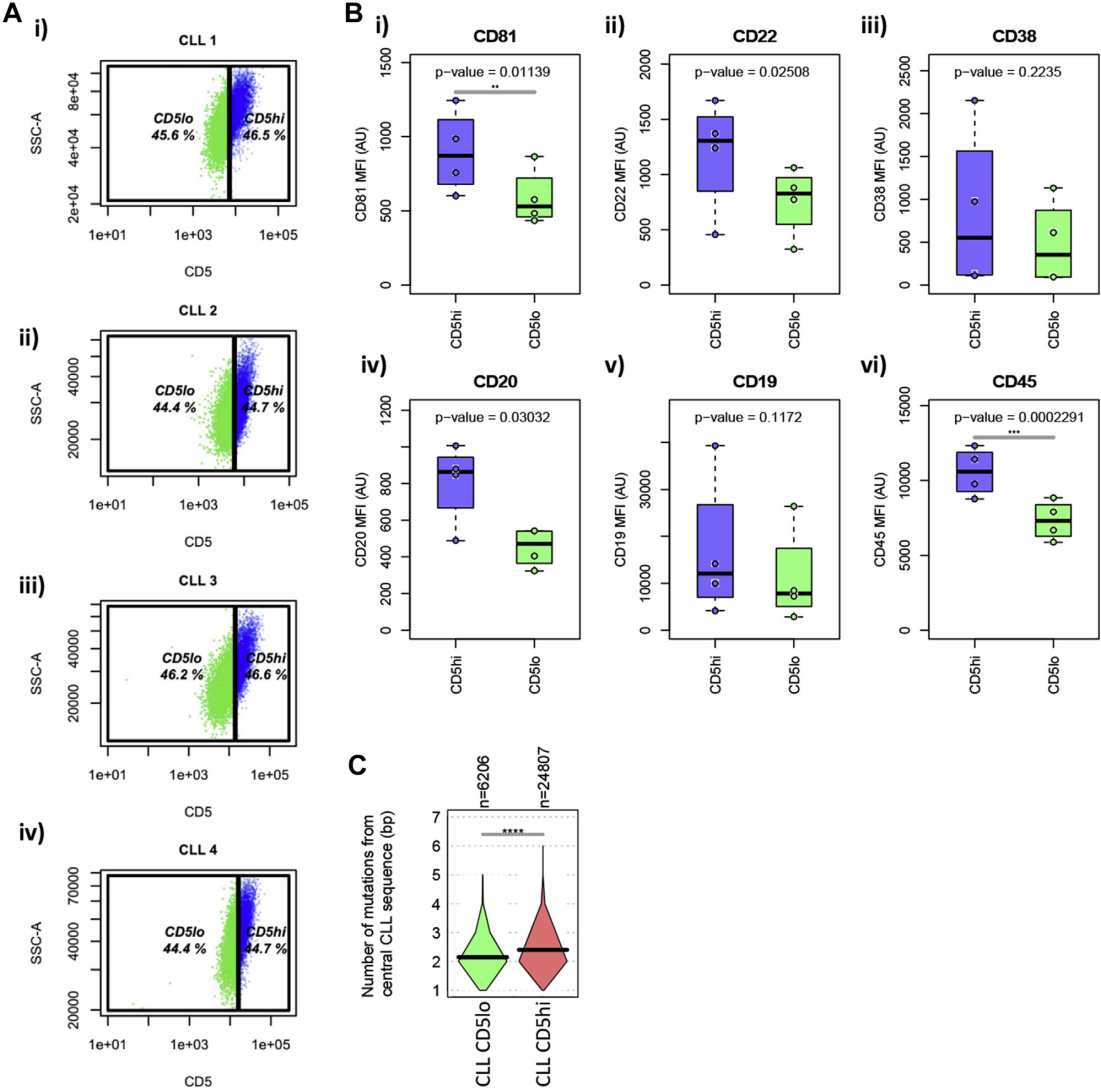
Testing the cell surface expression differences between CD5^high^ and CD5^low^ from 4 CLL patients. (**A**) The gating strategy used for identification of CD5^high^ and CD5^low^ peripheral blood B cells from PBMC samples, where CD5^high^ cells were defined as greater than 1.05 × (median CD5 fluorescence in the CD19^+^CD45^+^ lymphocytes, green gate) and CD5^low^ were defined as less than 0.95 × (median CD5 fluorescence in the CD19^+^CD45^+^ lymphocytes, blue gate). (**B**) The corresponding boxplots of the mean of fluorescence (MFI) between the CD5^high^ and CD5^low^ B-cell populations for (**i**) CD81, (**ii**) CD22, (**iii**) CD38, (**iv**) CD20, (**v**) CD19, and (**vi**) CD45. *p* values were calculated as paired two-tailed *t*-tests. (**C**) Violin plots of the numbers of mutations in the CD5^high^ and CD5^low^ BCR sequences from the sequencing data in Figure 2. **** *p* value < 10^−6^. Flow-cytometric fluorescence intensities of fluorophore-conjugated antibodies against a set of CLL-associated B-cell surface antigen (namely CD81, CD22, CD38, CD20, CD19, and CD45) were used to quantify cell-surface expression in the CLL clone. Notably, the mean of fluorescence (MFI) for the expression of both CD45 and CD81 by circulating CLL B cells was significantly lower for CD5^low^ B cells compared to CD5^high^ B cells (paired *t*-test *p* values of 0.011 and 0.00023, respectively; Supplementary Fig. E7). CD45 is normally found on all leukocytes, where previous studies show CD45 plays a positive regulatory role during B-cell receptor signaling [13]. CD81 is usually expressed at high levels in normal germinal center B cells, and associated with mutated CLL. This is in agreement with our data, as the CD5^high^ CLL B cells are associated with a higher number of mutations within the CLL clone as well as higher CD81 levels.

Our study shows that CD5^high^ and CD5^low^ tumor B cells were present in all CLL patients studied and displayed very similar population structures as determined by the phylogeny of their BCR repertoires. The overlapping BCR repertoires between CD5^high^ and CD5^low^ cells from CLL blood cells show that the disease encompasses a continuum of CD5 expression levels. This indicates that the CD5 state of CLL cells is subject to changes in CD5 expression and the observed dominance of CD5^high^ cells represents an equilibrated flux rather than a fixed state. Indeed, if the CD5^low^ CLL B-cell population were hierarchically above (i.e., closer to or containing stem cells) the CD5^high^ population, then the CD5^low^ CLL B-cell population would be a more effective treatment target than CD5^high^. Alternatively, if the CD5^low^ CLL B-cell population is hierarchically below the CD5^high^ CLL B-cell population (i.e., closer to differentiation and/or apoptosis), then understanding how CD5 expression can become downregulated may represent a therapeutic approach. Indeed, we show that decreased CD5 expression is associated with differences in CD81 and CD45 cell surface expression (Supplementary Fig. E7, online only, available at www.exphem.org), which may reflect biological differences between these groups of CLL cells. Together, these data suggest that CD5^low^ B cells are an important component of CLL that may be able to propagate and act as a residual disease population, with implications for understanding CLL pathogenesis, minimal residual disease, and developing anti-CD5-directed therapies.

## Figures and Tables

**Supplementary Table E1:** Patient sample information

Patient	White blood cell count (×10^9^ cells/L)	CLL Binet Stage	Treatment status
CLL 1	70.3	A	Untreated
CLL 2	102.4	C	Untreated
CLL 3	95.3	A	Chlorambucil, rituximab completed 2 years prior to sampling
CLL 4	69.1	C	Untreated
Healthy individuals 1–4	Healthy range	NA	NA

NA = not applicable.

**Supplementary Table E2:** Patient clinical information

Patient	Genomic abnormality	IgHV mutation status (% identity, bp differences)	Progression of disease
CLL 1	13q14.3 deletion	Mutated (96.6%, 7bp)	Progressive, chlorambucil then relapse, bendamustine/rituximab responder.
CLL 2	13q deletion	Unmutated (99.1%, 2 bp)	Progressive at time of sampling, chlorambucil then relapse, then rituximab/chlorambucil nonresponder.
CLL 3	Bi-allelic 13q deletion	Unmutated (99.5%, 1 bp)	Partial remission at time of sampling after rituximab/chlorambucil, went onto idelalisib/rituximab after sampling, but relapsed.
CLL 4	Not detected	Mutated (94.2%, 11bp)	Progressive at time of sampling, rituximab nonresponder.

Patients were selected for this study presented with a range of different prognostic factors and follow up information to ensure that the trends observed here could be generalized to multiple CLL subtypes. CLL 1, 2, and 3 exhibited 13q deletions, which are associated with good prognosis [1], and CLL 4 showed no known genomic abnormalities. A further prognostic factor is the IgHV mutation status, where unmutated IgHV (displayed in CLL 1 and 4) has a significantly inferior prognosis to mutated IgHV (displayed in CLL 2 and 3).

**Supplementary Table E3:** Patient clinical information

Marker type	Genomic/chromosomal markers	Relative prognosis	Reference
Deletions	Deletions in 11q, 17p	Poor	[1]
Deletions	Deletions in 13q	Good	[1]
Deletions	Deletion in 6q	Intermediate	[2]
Mutations	TP53, ATM (tumor suppressor genes)	Poor	[3]
Mutations	IRF4, Bcl-2 polymorphism	Good	[4]
Mutations	Bcl-6 mutation	Poor	[5]
Mutations	MDM2 SNP	Poor	[6]
IgVH mutational status	IgVH mutated	Good	7, 8, 9, 10
	IgVH unmutated	Poor	
Gene expression	ZAP-70 (correlates with mutational status)	Poor	[1]
	V3-21 gene usage	Poor	[1]
MicroRNAs	MicroRNA signature associated with prognosis	-	[11]
Telomere length	Longer telomere length (correlates with mutational status)	Good	[12]

**Supplementary Table E4:** Percentage of CD5^high^ and CD5^low^ B cells of total B cells in CLL and healthy patients’ peripheral blood

	Single-cell experiment		Bulk-cell experiment		Total B cells∗
	CD5^low^	CD5^high^	CD5^low^	CD5^high^	
CLL 1	1.551	98.449	-	-	6381
CLL 2	3.208	96.792	-	-	6826
CLL 3	2.968	97.032	-	-	6975
Healthy individual 1	95.376	4.624	-	-	757
Healthy individual 2	92.580	7.420	-	-	283
CLL 4	-	-	1.751	98.249	9993
Healthy individual 3	-	-	92.837	7.163	2080
Healthy individual 4	-	-	94.315	5.685	1970

∗ Total number of sorted CD19^+^CD20^+^CD5^+/−^ B cells.

**Supplementary Table E5:** Percentage of reads from the maximum BCR clone in the CD5^high^ and CD5^low^ B-cell populations in CLL and healthy individuals

	Sequencing method	% CLL sequences∗		Total number of sequences†	
		CD5^low^	CD5^high^	CD5^low^	CD5^high^
CLL 1	Single-cell	76.56	ND	64	ND
CLL 2	Single-cell	96.34	100.00	82	80
CLL 3	Single-cell	98.15	97.62	54	42
CLL 4	Bulk-cell	68.80	99.85	109775	397469
Healthy individual 3	Bulk-cell	NA	NA	206201	149673
Healthy individual 4	Bulk-cell	NA	NA	194555	11375

ND = not determined; NA = not applicable.

∗ Sequences within 5bp of sequences in the largest cluster.

† Single-cell sequencing performed by Sanger sequencing and bulk-cell sequencing performed by MiSeq.

**Supplementary Table E6:** Maximum cluster sizes as a percentage of total BCR reads for CLL 4, healthy individual 3, and healthy individual 4

	Maximum cluster size (% of total network)	
	CD5^high^ B cells	CD5^low^ B cells
CLL 4	99.85	68.8
Healthy individual 3	4.62	2.19
Healthy individual 4	21.17	3.1

**Supplementary Table E7:** Probabilities of BCR repertoire overlap between CD5^high^ and CD5^low^ populations in patient CLL 4 occurring by chance given a single switch event

Distance from central BCR	Number of CD5^low^ BCRs	BCRs observed in both CD5^low^ and CD5^high^ populations	Number of CD5^high^ BCRs	*p* value of observed overlap
1	613	581	584	<10 × 10^–10^
2	3749	522	714	<10 × 10^–10^
3	486	47	69	<10 × 10^–10^
4	28	1	3	1.05 × 10^–8^
5	1	1	2	<10 × 10^–10^
